# Financing for pandemic preparedness and response measures: a systematic scoping review

**DOI:** 10.2471/BLT.23.290207

**Published:** 2024-02-22

**Authors:** Roberto Duran-Fernandez, Daniel Bernal-Serrano, Jose Alberto Garcia-Huitron, Raymond Hutubessy

**Affiliations:** a Tecnológico de Monterrey, Escuela de Gobierno y Transformación Pública, Eugenio Garza Lagüera y, Av. Rufino Tamayo, Valle Oriente, San Pedro Garza García 66269, Mexico.; b Independent researcher, Washington DC, United States of America.; c Immunization, Vaccines and Biologicals, World Health Organization, Geneva, Switzerland.

## Abstract

**Objective:**

To obtain insights into reducing the shortfall in financing for pandemic preparedness and response measures, and reducing the risk of another pandemic with social and economic costs comparable to those of the coronavirus disease.

**Methods:**

We conducted a systematic scoping review using the databases ScienceDirect, Scopus, JSTOR, PubMed® and EconLit. We included articles published in any language until 1 August 2023, and excluded grey literature and publications on epidemics. We categorized eligible studies according to the elements of a framework proposed by the World Health Organization Council on the Economy of Health for All: (i) root/structural causes; (ii) social position/foundations; (iii) infrastructure and systems; and (iv) communities, households and individuals.

**Findings:**

Of the 188 initially identified articles, we included 60 in our review. Most (53/60) were published after 2020, when academic interest had shifted towards global financing mechanisms. Most (37/60) addressed two or more of the council framework elements. The most frequently addressed element was infrastructure and systems (54/60), discussing topics such as health systems, financial markets and innovation ecosystems. The roots/structural causes were discussed in 25 articles; communities, households and individuals in 22 articles; and social positions/foundations in 11.

**Conclusion:**

Our review identified three important gaps: a formal definition of pandemic preparedness and response, impeding the accurate quantification of the financing shortfall; research on the extent to which financing for pandemic preparedness and response has been targeted at the most vulnerable households; and an analysis of specific financial instruments and an evaluation of the feasibility of their implementation.

## Résumé

**Objectif:**

Mieux comprendre comment lutter contre le manque de moyens alloués aux mesures de préparation et de riposte aux pandémies, et comment réduire le risque d'une nouvelle pandémie aux coûts économiques et sociaux comparables à ceux engendrés par la maladie à coronavirus.

**Méthodes:**

Nous avons effectué une revue systématique exploratoire dans les bases de données ScienceDirect, Scopus, JSTOR, PubMed® et EconLit. Nous avons inclus les articles rédigés dans toutes les langues et publiés avant le 1^er^ août 2023, mais écarté la littérature grise et les publications relatives aux épidémies. Nous avons classé les études retenues en fonction des éléments d'un cadre soumis par le Conseil de l'Organisation mondiale de la Santé sur l'économie de la santé pour tous: (i) causes profondes/structurelles; (ii) positions/fondements sociaux; (iii) infrastructures et systèmes; et enfin, (iv) communautés, ménages et individus.

**Résultats:**

Sur les 188 articles identifiés initialement, nous en avons sélectionné 60 pour notre revue. La plupart (53/60) ont été publiés après 2020, quand l'intérêt académique s'est tourné vers les mécanismes de financement mondiaux. Une grande partie (37/60) des articles mentionnaient au moins deux éléments du cadre du Conseil. L'élément le plus souvent abordé était celui des infrastructures et systèmes (54/60), avec des thèmes tels que les systèmes de santé, les marchés financiers et les écosystèmes d'innovation. Les causes profondes/structurelles étaient évoquées dans 25 articles; les communautés, ménages et individus dans 22 articles; et les positions/fondements sociaux dans 11 articles.

**Conclusion:**

Notre revue a permis d'identifier trois grands absents: la définition officielle pour la préparation et la riposte aux pandémies, ce qui empêche de quantifier le manque de moyens avec précision; les recherches pour déterminer dans quelle mesure le financement de la préparation et de la riposte aux pandémies a ciblé les ménages les plus vulnérables; et pour terminer, une analyse des instruments financiers spécifiques et une évaluation consacrée à la faisabilité de leur mise en œuvre.

## Resumen

**Objetivo:**

Comprender mejor cómo combatir la falta de recursos asignados a las medidas de preparación y respuesta ante una pandemia, y cómo reducir el riesgo de una nueva pandemia con costes económicos y sociales comparables a los que causó el coronavirus.

**Métodos:**

Se realizó una revisión sistemática de alcance mediante las bases de datos ScienceDirect, Scopus, JSTOR, PubMed® y EconLit. Se incluyeron artículos publicados en cualquier idioma hasta el 1 de agosto de 2023, y se excluyó la literatura gris y las publicaciones sobre epidemias. Se categorizaron los estudios elegibles según los elementos de un marco propuesto por el Consejo sobre la Economía de la Salud para Todos de la Organización Mundial de la Salud: (i) causas fundamentales/estructurales; (ii) posiciones/fundamentos sociales; (iii) infraestructura y sistemas; y (iv) comunidades, hogares e individuos.

**Resultados:**

De los 188 artículos identificados en un principio, se incluyeron 60 en la revisión. La mayoría (53/60) se publicaron después de 2020, cuando el interés académico se había orientado hacia los mecanismos de financiación mundial. La mayoría (37/60) abordaban dos o más de los elementos del marco del Consejo. El elemento abordado con más frecuencia fue la infraestructura y el sistema (54/60), en los que se trataban temas como los sistemas sanitarios, los mercados financieros y los ecosistemas de innovación. Las causas fundamentales/estructurales se trataron en 25 artículos; las comunidades, hogares e individuos en 22 artículos; y las posiciones/fundamentos sociales en 11.

**Conclusión:**

La revisión identificó tres deficiencias importantes: una definición formal de preparación y respuesta ante una pandemia, lo que impide cuantificar con precisión el déficit de financiación; una investigación sobre el grado en que la financiación de la preparación y respuesta ante una pandemia se ha dirigido a los hogares más vulnerables; y un análisis de los instrumentos financieros específicos y una evaluación de la viabilidad de su implementación.

## ملخص

**الغرض:**

الحصول على رؤى متعمقة عن الحد من العجز في تمويل تدابير الاستعداد للجوائح والاستجابة لها، والحد من مخاطر حدوث جائحة أخرى ذات نتائج اجتماعية واقتصادية مماثلة لتلك الناجمة عن مرض فيروس كورونا.

**الطريقة:**

قمنا بإجراء مراجعة منهجية عن كثب باستخدام قواعد البيانات ScienceDirect، وScopus، وJSTOR، وPubMed®‎، وEconLit. وقمنا بتضمين المقالات المنشورة بأية لغة حتى 1 أغسطس/آب 2023، واستبعاد الأدبيات والمنشورات غير الرسمية المتعلقة بالأوبئة. قمنا بتصنيف الدراسات المؤهلة وفقًا لعناصر إطار العمل المقترح بواسطة مجلس منظمة الصحة العالمية، والمختص باقتصاد الصحة للجميع (1) :(Economy of Health for All) الأسباب الجذرية/الهيكلية؛ و(2) الوضع الاجتماعي/الأسس الاجتماعية؛ و(3) البنية التحتية والأنظمة؛ و(4) المجتمعات والأسر والأفراد.

**النتائج:**

من بين 188 مقالة تم تحديدها بشكل مبدئي، قمنا بتضمين 60 منها في مراجعتنا. تم نشر معظمها (53/60) بعد عام 2020، عندما تحول الاهتمام الأكاديمي نحو آليات التمويل العالمية. وتناولت معظم هذه المقالات (37/60) عنصرين أو أكثر من عناصر إطار العمل التابع للمجلس. وكان العنصر الأكثر تناولًا هو البنية التحتية والنظام (54/60)، حيث شمل مناقشة موضوعات مثل الأنظمة الصحية، والأسواق المالية، والنظم البيئية للابتكار. كذلك، تمت مناقشة الأسباب الجذرية/الهيكلية في 25 مقالة؛ ومناقشة المجتمعات، والأسر، والأفراد في 22 مقالة؛ ومناقشة الأوضاع/الأسس الاجتماعية في 11 مقالة.

**الاستنتاج:**

أشارت مراجعتنا إلى ثلاث فجوات مهمة، وهي: التعريف الرسمي للاستعداد للجوائح والاستجابة لها، مما يعوق القياس الكمي الدقيق للعجز في التمويل؛ والبحث عن مدى استهداف تمويل الاستعداد للجوائح والاستجابة لها، للأسر الأكثر تعرضًا للخطر؛ وتحليل أدوات مالية محددة وتقييم جدوى تنفيذها.

## 摘要

**目的:**

深入了解如何减少大流行病防范和应对措施的资金短缺的情况，并降低再次发生与新型冠状病毒肺炎疫情社会和经济成本相当的大流行病的风险。

**方法:**

我们使用 ScienceDirect、Scopus、JSTOR、PubMed® 和 EconLit 数据库进行了系统性的范围综述。我们纳入了截至 2023 年 8 月 1 日以任何语言发表的文章，并排除了有关流行病的灰色文献和出版物。我们根据世界卫生组织理事会提出的全民健康经济框架的要素对符合条件的研究进行了分类：(i) 根本原因/结构性原因；(ii) 社会地位/基础；(iii) 基础设施和系统；(iv) 社区、家庭和个人。

**结果:**

在最初确定的 188 篇文章中，我们在综述中纳入了 60 篇。大部分文章 (53/60) 是在 2020 年之后发表的，当时学术兴趣已转向全球筹资机制。大部分文章 (37/60) 涉及两个或多个理事会框架要素。最常被提及的要素是基础设施和系统 (54/60)，讨论了卫生系统、金融市场和创新生态系统等主题。有 25 篇文章讨论了根本/结构性原因要素；有 22 篇文章讨论了社区、家庭和个人要素；有 11 篇文章讨论了社会地位/基础要素。

**结论:**

我们的综述发现了三个重要缺口：对大流行病防范和应对的正式定义，使我们难以准确量化资金短缺的情况；研究为大流行病防范和应对筹措的资金在多大程度上用于最弱势家庭；对具体金融工具的分析及其实施可行性的评估。

## Резюме

**Цель:**

Получить представление о том, как сократить дефицит финансирования мер по обеспечению подготовленности к пандемии и реагированию на нее, а также снизить риск возникновения еще одной пандемии с социальными и экономическими издержками, сопоставимыми с последствиями коронавирусной инфекции.

**Методы:**

Был проведен систематический обзор с использованием баз данных ScienceDirect, Scopus, JSTOR, PubMed® и EconLit. В исследование были включены статьи, опубликованные на любом языке до 1 августа 2023 года, и исключены серая литература и публикации об эпидемиях. Соответствующие требованиям исследования были классифицированы в соответствии с элементами структуры, предложенной Советом Всемирной организации здравоохранения по экономике здоровья для всех: (i) глубинные/структурные причины; (ii) социальное положение или основы; (iii) инфраструктура и системы; (iv) общины, домохозяйства и отдельные люди.

**Результаты:**

Из 188 первоначально выявленных статей в обзор вошли 60. Большинство из них (53/60) было опубликовано после 2020 года, когда научный интерес сместился в сторону глобальных механизмов финансирования. Большинство из них (37/60) затрагивало два или более элемента рамочного решения Совета. Чаще всего рассматривался такой элемент, как инфраструктура и система (54/60), при этом обсуждались такие темы, как системы здравоохранения, финансовые рынки и инновационные экосистемы. Глубинные/структурные причины обсуждались в 25 статьях; общины, домохозяйства и отдельные люди – в 22 статьях; социальные позиции или основы – в 11.

**Вывод:**

В ходе обзора были выявлены три важных пробела: официальное определение подготовленности к пандемии и ответных мер, что препятствует точной количественной оценке дефицита финансирования; исследование масштабов финансирования готовности к пандемии и ответных мер, направленных на наиболее уязвимые домохозяйства; анализ конкретных финансовых инструментов и оценка возможности их применения.

## Introduction

The coronavirus disease 2019 (COVID-19) pandemic has been one of the deadliest emergencies in modern history, with a global death toll exceeding 14.9 million people.^1^ Beyond the tragic loss of life, the pandemic has wrought staggering economic damage. Some studies estimate its impact at 16 trillion United States dollars (US$) in the United States of America alone.^2^

To reimagine the relationship between economics and health, the World Health Organization (WHO) Council on the Economics of Health for All was established during the COVID-19 pandemic in November 2020 by the WHO Director-General. The council was tasked with recommending specific policy approaches to bring health for all to the heart of government decision-making, public–private alliances and global collaboration.^3^ The council acknowledged the complexity of health and its interdependence on a diverse array of factors – including economic, social, environmental and financial dimensions – and proposed a framework that is based on measuring the value of health and well-being as opposed to measuring the price of everything.^4^^,^^5^ Built on a foundation of theoretical and policy research that has explored the interplay between economic factors, health and the determinants of health,^6^^–^^8^ this framework is based on the elements: (i) roots/structural causes; (ii) social positions/foundations; (iii) infrastructure and systems; and (iv) communities, households and individuals (a schematic figure of the framework has been published elsewhere).^4^

During the period of the council’s research, the World Bank and WHO identified an annual investment need of US$ 31.1 billion for a global pandemic preparedness and response system.^9^ Understanding financing for pandemic preparedness and response at a global level is now more urgent than ever. However, a comprehensive literature synthesis on the mechanisms to prepare, adapt and respond to the economic demands of a pandemic is needed, and the international community has yet to establish a comprehensive financing framework to manage future outbreaks.^10^

We therefore undertook a systematic scoping review of the academic literature to characterize evidence on the strategies, successes, challenges and opportunities to address pandemics from a financial standpoint.^11^ Our aim was to obtain insights into reducing the shortfall in financing for pandemic preparedness and response, and the risk of another pandemic with social and economic costs comparable to those of COVID-19. Because pandemics are characterized for their long-term consequences and their complex, multidimensional, multifaceted nature that extends beyond the immediate reach of the health sector,^12^ we adopt the council’s framework to characterize the academic literature on financing pandemic preparedness and response measures. 

## Method

### Database search

We registered our review on the International Platform of Registered Systematic Review and Meta-analysis Protocols (INPLASY202380111),^13^ and followed Preferred Reporting Items for Systematic Reviews and Meta-Analyses guidelines for Scoping Reviews.^14^^,^^15^

We conducted a three-stage search for relevant publications across ScienceDirect, Scopus, JSTOR, PubMed® and EconLit, focusing on the keywords: “pandemic”, “preparedness”, “response”, “financing”, “finance” and “funding.” In ScienceDirect, Scopus and JSTOR, we targeted research articles, review articles and discussions with the strategies: (i) “pandemic preparedness and response” AND (“financing” OR “finance” OR “funding”); and (ii) “pandemic prevention preparedness and response” AND (“financing” OR “finance” OR “funding”). We also ran these search strategies in French and Spanish, but without the financing and related keywords filter. For PubMed®, we included all papers found without applying finance-related filters. The database EconLit required a more flexible search strategy, in which keywords were sought in any order of appearance. 

### Eligibility criteria

Our search was restricted to articles published from 1900 until 1 August 2023, with no language restrictions. We focused on peer-reviewed research articles on the financing of pandemic preparedness and response measures at the global and regional levels, excluding grey literature such as reports, policy documents, working papers, newsletters, government documents, speeches, book chapters, letters, white papers, guidelines and protocols. We also excluded studies focused solely on single countries. Because of the qualitative difference between pandemics and epidemics, we also excluded studies describing financing for epidemic preparedness and response. 

### Data extraction

We created a spreadsheet database to record the bibliographical details of each paper and copied the abstract of each paper into the spreadsheet. One author read the full text of each paper to assess eligibility, determining whether the focus was financing for pandemic preparedness and response and whether the geographical eligibility criteria were met. The same author copied specific sentences of high relevance from each paper into the spreadsheet.

We used the consolidated criteria for reporting qualitative research checklist to assess the quality and relevance of the study to our research question (how has scientific literature addressed the multifaceted aspects of financing for pandemic preparedness and response at the global level?).^16^ To ensure objectivity and accuracy, all four authors independently reviewed the quality assessment of each article. To assess the quality of the journals in which the articles were published, we used the Scimago journal and country rank tool (Scimago, Granada, Spain).^17^

We then analysed the general characteristics of each study; one author studied the full-text documents, focusing on the discussion sections, to extract the primary findings of each paper. The same author used these data to classify the studies according to one or more of the elements of the preliminary framework proposed by the WHO Council on the Economics of Health for All.^4^ Another author independently reviewed the eligibility criteria and initial classifications for each author, referencing the database and summary tables; another author provided oversight and feedback on the results. Where we considered the database and summary tables to lack sufficient depth, we re-examined the full text of the paper. We resolved disagreements with regards to framework element classification by majority consensus.

## Results

**Fig. 1:**
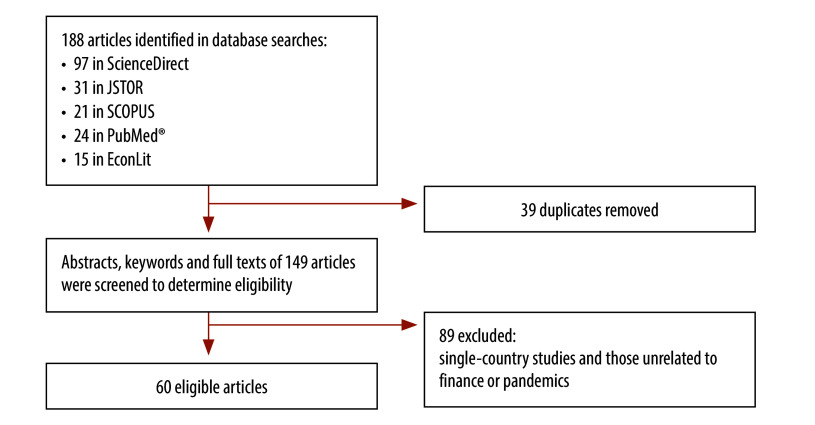
Selection of publications for inclusion in systematic scoping review on financing of pandemic preparedness and response measures

We identified 188 records: 97 in ScienceDirect, 31 in JSTOR (articles and reviews), 21 in SCOPUS, 24 in PubMed® and 15 in EconLit. After excluding duplicates, we identified 149 unique publications (Fig. 1). Our final review considered 60 articles (Table 1; available at: https://www.who.int/publications/journals/bulletin/).^18^^–^^77^ We assessed the quality of the journals (average h-score of 147):^78^ 56 (93.3%) articles were published in Q1 journals and 4 (6.7%) articles in Q2 journals.

**Table 1:** Properties of reviewed publications on global financing for pandemic preparedness and response measures, categorized according to the four elements of the framework proposed by the World Health Organization (WHO) Council on the Economics of Health for All^4^

Author/year	Article type	Study aim	Element of framework proposed by the WHO Council on the Economics of Health for All^4^			
			Root/ structural causes (25 articles)	Social position/ foundations (11 articles)	Infrastructure and system (54 articles)	Communities, households and individuals (22 articles)
Daems et al., 2005^18^	Research	To identify challenges in pandemic vaccine development, including planning and coordination, and propose public–private partnership models^a^	No	No	Yes	Yes
Gostin & Berkman, 2007^19^	Research	To identify the legal and regulatory aspects of pandemic preparedness and response measures, including financial aspects such as liabilities and compensations derived from such measures	No	No	Yes	No
Ijsselmuiden et al., 2008^20^	Discussion	To examine global investments in health research, and the spending gap between developed and developing nations, and propose new research priorities in health, including pandemic preparedness, climate change and sustainable financing	Yes	No	No	Yes
Ortu et al., 2008^21^	Research	To examine the legal aspects of pandemic preparedness and response measures, including insurance and workers’ compensation	No	No	Yes	No
Yen et al., 2015^22^	Review	To highlight the role of funding in vaccine stockpiles and discuss public policy measures for emergencies	No	No	Yes	No
Katz & Seifman, 2016^23^	Discussion	To highlight the importance of sustained support for pandemic preparedness, especially in low-income countries, and identify funding mechanisms^a^	Yes	Yes	No	Yes
Leigh et al., 2018^24^	Research	To examine existing international funding mechanisms, such as the WHO Contingency Fund for Emergencies and Coalition for Epidemic Preparedness and Innovations, and highlight the importance of adequate funding in pandemic preparedness and response measures planning	No	No	Yes	No
Babu et al., 2021^25^	Review	To examine the importance of investment in resilient health systems in Afghanistan, Bangladesh, Bhutan, India, Maldives, Nepal, Pakistan and Sri Lanka, as well as planning and best-practice adoption	No	No	Yes	No
Charani et al., 2021^26^	Review	To examine investment and policies related to antimicrobial resistance, in terms of both government and the private sector	Yes	No	Yes	No
Duff et al., 2021^27^	Review	To investigate the need for a renewed framework for global collective action that ensures conformity with international regulations, and promotes effective prevention and response to pandemic infectious diseases; to propose recommendations to strengthen global public health governance^a^	No	No	Yes	No
Giersing et al., 2021^28^	Review	To highlight the importance of financial incentives and stakeholder collaboration in vaccine innovations	No	No	Yes	No
Kleinert & Horton, 2021^29^	Discussion	To highlight the rapid need for diagnostic capacity and testing, and for equitable access to diagnostics	No	No	Yes	No
Lal et al., 2021^30^	Review	To examine the fragmented governance in pandemic response with separate funding streams and varying priorities; to call for integration of global health security into UHC^a^	Yes	No	Yes	Yes
Lurie et al., 2021^31^	Review	To highlight the importance of a global research and development financing system in pandemic preparedness, including political challenges	Yes	No	Yes	No
Sirleaf & Clark, 2021^32^	Discussion	To highlight the importance of funding in addressing gaps and preventing future disease outbreaks; to recommend the establishment of an International Pandemic Financing Facility	No	No	Yes	No
Agarwal & Reed, 2022^33^	Research	To analyse COVID-19 vaccine advance purchase agreements, and propose financial alternatives to absorb risk and strengthen the financing of pandemic preparedness and response measures^a^	No	Yes	Yes	Yes
Akenroye et al., 2022^34^	Research	To analyse the main drivers of and barriers to the effective implementation of a collaborative model of pandemic preparedness and response	Yes	No	Yes	No
Bollyky et al., 2022^35^	Research	To highlight the importance of investments in risk communication strategies to foster public confidence	Yes	No	Yes	No
Carlson & Phelan, 2022^36^	Review	To investigate the need for capacity-building and investment to support the broader One Health approach to disease surveillance and outbreak prevention, including financing and technology transfer	No	Yes	Yes	No
Cernuschi et al., 2022^37^	Research	To examine government intervention in vaccine markets and the possibility of regional shared manufacturing facilities, as well as improved access and affordability for all countries	No	No	Yes	Yes
Clark et al., 2022^38^	Discussion	To highlight the benefits of stronger international leadership and increased financing for the Access to COVID-19 Tools Accelerator; to emphasize the importance of transparent financing and agreements to ensure equitable distribution of vaccines and other medical tools^a^	No	No	Yes	No
Frenk et al., 2022^39^	Discussion	To emphasize the importance of sustainable development and robust financing for global health security, and propose mandatory contributions by Member States	Yes	No	No	No
Gwenzi et al., 2022^40^	Review	To examine financial tools for local funding in low-income countries for zoonotic disease research and mitigation, proposing specific instruments such as taxes and industry-specific levies in areas of livestock and wildlife tourism^a^	Yes	No	No	No
Hayman et al., 2022^41^	Research	To investigate vaccine manufacturing aspects, challenges, investments in research and development, technology and multistakeholder coordination^a^	No	No	Yes	No
Lal et al., 2022^42^	Review	To emphasize the need to integrate investments in health systems to strengthen both global health security and UHC; to propose a Pandemic Fund^a^	No	No	Yes	Yes
Meier et al., 2022^43^	Review	To examine international legal reforms and new treaties related to pandemic preparedness and response measures, focusing on financing mechanisms, equity, social justice and human rights in the global health system^a^	Yes	No	Yes	Yes
Moeti et al., 2022^44^	Review	To highlight the importance of investing in research, development and preparedness for health emergencies, stressing the importance of equitable distribution of vaccines as well as synergy between the various global health agendas^a^	No	Yes	Yes	Yes
Olliaro & Torreele, 2022^45^	Review	To examine the limitations in research and development, and emphasize the shift towards treating health interventions as a common good and the need for public investments to overcome market failures	No	No	Yes	Yes
Reid-Henry et al., 2022^46^	Discussion	To propose a global public investment for international finance, focusing on transparency, coordination, co-creation and consultation, to reimagine pandemic preparedness and response measures^a^	No	Yes	Yes	Yes
Ren et al., 2022^47^	Research	To highlight the importance of access to antibiotics in pandemics, emphasizing the need for coordinated financing mechanisms and equitable resource allocation, including incentives for research and development, transparent markets and global partnerships	No	Yes	Yes	Yes
Rosa et al., 2022^48^	Research	To emphasize the importance of research investments in palliative care in low- and middle-income countries, and call for the removal of various barriers to health care	Yes	Yes	Yes	No
Sachs et al., 2022^49^	Review	To highlight the need for a Global Health Fund aligned with WHO to expand existing health funds, providing financing for disease control and health system strengthening in low- and middle-income countries^a^	No	No	Yes	Yes
Saxenian et al., 2022^50^	Research	To investigate sustainable financing for immunization, and to emphasize the need to prioritize adequate funding, efficient service delivery and the right combination of revenue-raising strategies; to highlight the role of governments and development partners in strengthening public financial management^a^	Yes	No	Yes	No
Sekalala & Rawson, 2022^51^	Research	To examine the pandemic treaty as a potential opportunity to address effective and equitable access to medical countermeasures by creating conditions for government-funded research and development	No	No	Yes	Yes
Solomon, 2022^52^	Discussion	To reflect on a global agreement for pandemic preparedness measures and emphasize pragmatic, creative solutions within limited timeframes^a^	No	No	Yes	No
Tacconelli et al., 2022^53^	Review	To examine the challenges and recommendations for improving global pandemic preparedness and response financing by examining collaborative research projects, highlighting the need for alternative funding formats and coordination	No	No	Yes	No
Williamson et al., 2022^54^	Discussion	To examine proposals to improve global governance in pandemic preparedness and response measures, proposing the Financial Intermediary Fund (later Pandemic Fund) and potential changes to IHR^a^	Yes	No	Yes	No
Akselord et al., 2023^55^	Discussion	To advocate increased health system investment and financing for equity, resilience and sustainability, highlighting UHC, global health architecture, gender equality and transformative change^a^	Yes	Yes	Yes	No
Archer et al., 2023^56^	Discussion	To emphasize the importance of enhancing disease surveillance and intelligence during public health emergencies; to propose financing to facilitate sustainable preparedness and the creation of National Investment Plans	No	No	Yes	No
Bochner et al., 2023^57^	Research	To examine the performance of health emergency systems, and propose increased investments at health facility and intermediate public health levels	No	No	Yes	No
Boyce et al., 2023^58^	Research	To examine emerging concerns about the Pandemic Fund, comparing it with the Pandemic Emergency Financing Facility^a^	No	No	Yes	No
Byanyima et al., 2023^59^	Discussion	To examine financing systems for community-led organizations, emphasizing the importance of overcoming funding barriers and integrating community data	No	No	Yes	Yes
Elnaiem et al., 2023^60^	Review	To highlight underinvestment in pandemic preparedness and response measures; to call for targeted investments in various areas, including climate change mitigation	Yes	No	Yes	No
Ford et al., 2023^61^	Review	To emphasize the importance of public–private partnerships, funding for research and development, regional vaccine manufacturing and community engagement^a^	No	No	Yes	Yes
Gallo-Cajiao et al., 2023^62^	Review	To examine the need for funding to establish governance structures and propose a Global Pandemic Financing Facility to reduce the risk of future pandemics^a^	Yes	No	Yes	No
Gostin et al., 2023^63^	Review	To advocate the incorporation of human rights into health emergency preparedness and response, emphasizing global health and rights architecture	Yes	No	Yes	Yes
Helldén et al., 2023^64^	Review	To analyse current funding trends for digital initiatives related to pandemic preparedness and response measures across major donors and development partners	No	No	Yes	No
Hiroshima G7 Global Health Task Force, 2023^65^	Discussion	To examine investments, public policy for health resilience, long-term research and development investments, and policy measures connecting health, resilience and climate change; to call for the integration of pandemic preparedness and response measures within UHC^a^	Yes	Yes	Yes	No
Kasaeva et al., 2023^66^	Discussion	To advocate the inclusion of One Health principles in the negotiations for a global Pandemic Instrument and to discuss the direction of the Pandemic Fund^a^	Yes	No	Yes	No
Katz, 2023^67^	Discussion	To emphasize funding challenges for pandemic preparedness and advocate standardized reporting; to propose considerations in the design of the Pandemic Fund	No	No	Yes	No
Khosla et al., 2023^68^	Discussion	To highlight issues in a Pandemic Accord draft, emphasize equitable financing, and raise concerns about power imbalance and the need for human rights and global health pathways^a^	No	No	Yes	No
Lee, 2023^69^	Discussion	To highlight issues around state-centric paradigms in the Pandemic Acord draft; to advocate inclusive decision-making and the rebuilding of trust in science and politics^a^	Yes	No	Yes	Yes
Micah et al., 2023^70^	Research	To examine the amount of development aid given for pandemic preparedness and response measures during the first 2 years of the COVID-19 pandemic, focusing on disparities between high- and low-income countries; to assess global health expenditure, development assistance and the ongoing necessity to maintain funding for essential global health functions	Yes	Yes	No	Yes
Morris, 2023^71^	Research	To consider the challenge of competing demands on World Bank resources to finance climate change and pandemic preparedness and response measures	Yes	No	No	No
Müller et al., 2023^72^	Discussion	To highlight the importance of surveillance as an evidence-based tool to understand population immunity and track viral transmission; to highlight the need for financing and investment in research infrastructure and data collection	No	No	Yes	Yes
Nardi et al., 2023^73^	Research	To address the role of financing, including regional organizations and financial institutions, in bolstering pandemic preparedness and response, particularly in low- and middle-income countries in the WHO Region of the Americas; to highlight challenges related to vaccine procurement and collaborative financial support and policy guidance	No	No	Yes	No
Saxena et al., 2023^74^	Review	To emphasize the relevance of good governance of the Pandemic Accord to guarantee equitable access to therapeutics and vaccines; to analyse the crucial role of investment in the development of research and development systems that reward pharmaceuticals while ensuring the availability of intellectual property rights^a^	Yes	No	Yes	No
Stubbs et al., 2023^75^	Review	To examine the challenge of securing increased national health budgets in low- and middle-income countries in the face of austerity measures driven by external debt, highlighting the need to change global rules on debt recovery and the importance of domestic resource mobilization	Yes	Yes	Yes	No
Torreele et al., 2023^76^	Discussion	To advocate the viewing of pandemic preparedness technologies as common goods, focusing on equity and knowledge sharing	Yes	No	Yes	Yes
Torreele et al., 2023^77^	Discussion	To emphasize the need for financing designed for health impact, proposing pre-negotiated agreements involving various sectors^a^	No	No	Yes	Yes

COVID-19: coronavirus disease 2019; IHR: International Health Regulations; UHC: universal health coverage; WHO: World Health Organization.

^a^ Paper proposed or discussed specific pandemic preparedness and response financing instruments.

We identified only seven articles published before 2020, which focused primarily on financial assistance to low-income countries, vaccine funding, and legal aspects of funding pandemic preparedness and response. The remaining 53 articles were published post-2020, after the start of COVID-19, when academic interest had shifted towards global financing mechanisms, including 20 studies on initiatives such as the Pandemic Fund and the establishment of new financial architectures under the WHO Pandemic Treaty negotiations. The scope of the post-2020 literature is generally limited to discussing specific elements of ongoing proposals, or analysis of policy or technical principles that underpin the strategy of financing for pandemic preparedness and response. Although the literature offers best practices supported by evidence and theory, objective assessments of the technical aspects, political viability, implementation feasibility and actual results are absent.

We identified 26 papers (Table 1) that propose or discuss specific pandemic preparedness and response financing instruments, including public policy tools, public–private partnerships and common good investment funding. Notably, discussions of proposals are mainly focused on two global funding mechanisms: the Pandemic Fund and the Access to COVID-19 Tools Accelerator. Five reviewed publications explore the relationship between universal health coverage (UHC) and global health security.^30^^,^^36^^,^^42^^,^^44^^,^^65^

With regards to the four elements of the framework proposed by the WHO Council on the Economics of Health for All, most reviewed publications (37; 61.7%) addressed two or more of these; the remainder (23; 38.3%) addressed a single element. The most frequently addressed element was infrastructure and system (54; 90.0%), followed by roots/structural causes (25; 41.7%), communities, households and individuals (22; 36.7%) and finally social positions/foundations (11; 18.3%). We discuss each of these below, as well as the issue of a lack of consistency in the literature in terminology.

### Infrastructure and system

Within this element of the council framework, addressed by 54 of the reviewed publications,^18^^,^^19^^,^^21^^,^^22^^,^^24^^–^^38^^,^^41^^–^^69^^,^^72^^–^^77^ topics such as health systems, financial markets and innovation ecosystems were frequently discussed (Table 1). The reviewed publications examine innovation, vaccine research and development, manufacturing, funding and capacity-building, emphasizing the need for a multifaceted approach towards strengthening global health systems and the pivotal role of governments in vaccine markets. The literature highlights a pressing need to reform global financial institutions and develop robust, adaptable financial frameworks. Central to these discussions is a call for increased investment in health systems to bolster resilience, coupled with the critical importance of effective risk communication strategies. The literature identifies significant obstacles (e.g. funding limitations and differences in organizational culture) that obstruct efficient collaboration between various stakeholders. Research suggests that an integrated approach is necessary, one that combines public institutes and private businesses. 

### Root/structural causes 

The 25 publications addressing this element of the council framework discussed topics such as governance, politics and economics;^20^^,^^23^^,^^26^^,^^30^^,^^31^^,^^34^^,^^35^^,^^39^^,^^40^^,^^43^^,^^48^^,^^50^^, ^^54^^,^^55^^,^^60^^,^^62^^,^^63^^,^^65^^,^^66^^,^^69^^–^^71^^,^^74^^–^^76^ 19 of these articles also explored aspects of the infrastructure and system element, although with a different focus (Table 1).^26^^,^^30^^,^^31^^,^^34^^,^^35^^,^^43^^,^^48^^,^^50^^,^^54^^,^^55^^,^^60^^,^^62^^,^^63^^,^^65^^,^^66^^,^^69^^,^^74^^–^^76^ For example, the discussion of new global collaboration mechanisms such as the Pandemic Fund is centred on governance. Other key topics discussed in the reviewed literature include the importance of sustainable development and robust financing for global health security (which involves navigating the challenge of competing demands between health and climate finance); financing pandemic preparedness and response in low-income countries; the role of international development agencies; and financial solutions for zoonotic disease research and mitigation. 

### Communities, households and individuals

The most frequently discussed topics within the 22 publications addressing this element were access to services and resources, and equity in access (Table 1).^18^^,^^20^^,^^23^^,^^30^^,^^33^^,^^37^^,^^42^^–^^47^^,^^49^^, ^^51^^,^^59^^,^^61^^,^^63^^,^^69^^,^^70^^,^^72^^,^^76^^,^^77^ The literature is focused on the need for cohesive and equitable strategies in pandemic preparedness and response, highlighting the importance of integrating global health security into UHC with an emphasis on human rights, equity and social determinants of health. A crucial topic is the need to understand the treatment of pandemic technologies, such as vaccines, as common goods, focusing on equity and knowledge sharing. Notably, 19 of these 22 articles also discuss topics within the infrastructure and system element.^18^^,^^30^^,^^33^^,^^37^^,^^42^^,^^43^^,^^45^^–^^47^^,^^49^^, ^^51^^,^^59^^,^^61^^,^^63^^,^^69^^,^^70^^,^^72^^,^^76^^,^^77^ This overlap includes discussions on financing systems and structures, public–private partnerships in research and development, and global health systems and security. 

### Social position/foundations 

Our review revealed that this framework element was addressed in the literature in only 11 articles, and only as a method of differentiating policies between countries of different levels of income (Table 1).^23^^,^^33^^,^^36^^,^^44^^,^^46^^–^^48^^,^^55^^,^^65^^,^^70^^,^^75^ There has been no research undertaken to analyse, for example, the distribution of financing for pandemic preparedness and response between individuals of different income levels in any given population. Only two articles discussed the educational dimension of financing for pandemic preparedness and response;^44^^,^^48^ education is a key sector that can play a crucial role in such systems, and a sector that was greatly affected by COVID-19 quarantines. Only one publication explored the effect of sex and gender on the receipt of finance for pandemic preparedness and response;^55^ the effect of occupation, ethnicity, race and financial literacy remain absent from the literature. Although equity in access to pandemic preparedness and response measures is an essential target, discussions on access by particularly vulnerable groups are not available in the literature.

### Terminology

Although the literature often refers to financing for pandemic preparedness and response, there is no unified definition of this wording. Of all 60 reviewed publications, only six attempt to define concepts before engaging in analysis and discussion.^20^^,^^27^^,^^30^^,^^56^^,^^62^^,^^67^ One publication deals with the definition of cooperative surveillance,^56^ which is only one element of pandemic preparedness and response; and others list the elements of a global public health system,^20^^,^^27^^,^^30^^,^^62^^,^^67^ potentially providing the basis for a definition. Our review also revealed a lack of consistency in the terminology. Both “pandemic preparedness and response” and “pandemic prevention, preparedness and response” have been widely used by international organizations and policy-makers in the COVID-19 response. Neither of these expressions have been officially ratified, and their meaning can vary with context.^79^^–^^81^

## Discussion

Our review has emphasized the need for a robust, equitable and sustainable global financial framework for pandemic preparedness and response, extending beyond the advocacy of a Pandemic Fund. The literature also highlights the importance of a comprehensive One Health approach, and enhanced disease surveillance systems for early detection and effective management of health emergencies. The reviewed publications advocate strengthening health systems, particularly in low- and middle-income countries, through increased investment as well as the integration of UHC and equity in access to health systems.

The literature highlights the need for improved collaborative models and global governance in pandemic preparedness and response, addressing barriers such as organizational cultural differences and funding constraints. Financial sustainability and equity remain central themes, with discussions on the necessity of mandatory contributions to global health funds and the development of new financing facilities. Our review also emphasizes the importance of legal and regulatory frameworks in pandemic response, and the need for continued investments in research and development (e.g. vaccine research and antimicrobial resistance). 

A strength of our review is our demonstration that the framework proposed by the WHO Council on the Economics of Health for All is instrumental in characterizing relevant literature, highlighting a focus on financing health systems and UHC, vaccine development and manufacture, and surveillance systems. However, such a focus is to the detriment of advancing knowledge on crucial health dimensions, including equity, access, and distributional impacts on minority or vulnerable groups. 

Our review had three main limitations. First, although the aim of our review is to improve our understanding of the international financial architecture for responding to future pandemics, the reviewed literature focuses on the terms that were broadly used in the COVID-19 response. This focus is a direct result of the key finding that the majority of existing academic literature on financing for pandemic preparedness and response was published in response to COVID-19. Authors may have referred to financing for pandemic preparedness and response using different terminology, especially in nonmedical literature and works published before 2020, potentially introducing biases. Furthermore, our review does not examine the well-established literature on health financing and sustainable development goal budgeting for health, which will include investments to mitigate the risks and costs of pandemics. 

Second, we excluded grey literature from our review. Although grey literature can provide valuable insights and inform decisions, concerns exist regarding the quality of the review process for grey literature, its accessibility and potential biases.^82^^–^^84^ The pandemic rapidly increased the volume and diversity of grey literature with unstable internet addresses, extending beyond traditional sources such as official reports to include journalistic pieces.^85^ The inclusion of grey literature would necessitate adapting existing protocols to address its unique characteristics, a task that falls outside the scope of this work. By focusing on peer-reviewed publications, we have followed an approved review process with a standardized quality evaluation based on journal rankings. Another advantage of only including peer-reviewed publications in our review is the existence of established retraction mechanisms for correcting inaccuracies. We recommend further research to allow the existing grey literature to be characterized and included in future reviews.

Third, we excluded literature on financing epidemics from our review. We acknowledge that omitting articles on epidemic preparedness may overlook strategies that are applicable to pandemics, even though they are distinct phenomena. Financing an epidemic, which typically presents a partial equilibrium challenge, can often be managed with traditional risk management approaches.^86^ In contrast, pandemics pose a significant threat to global health security with long-term consequences.^12^^,^^76^ The required investment in global commons encompasses shared international structures that enhance global health security, including aspects of trade, transportation, the environment and access to medicines, particularly for developing countries during emergencies.^87^ Pandemics have widespread effects that complicate the basic assumptions used in simpler economic models, which typically assume that all other economic conditions remain unchanged.^88^^,^^89^

Our review has identified three important gaps in the literature: an accurate quantification of the financing shortfall is impeded by the lack of a formal definition of pandemic preparedness and response; although policy papers suggest that the COVID-19 pandemic disproportionately affected vulnerable households, there are currently no publications analysing the extent to which financing for pandemic preparedness and response has been targeted at such households;^90^ and there is currently no analysis of specific financial instruments except for those proposed by international financial institutions such as the World Bank and International Monetary Fund,^80^^,^^91^ and no evaluation of the political and administrative feasibility of their implementation. Addressing these gaps in future research is an important step towards achieving adequate global financing instruments for pandemic preparedness and response measures.

## References

[1] 14.9 million excess deaths associated with the COVID-19 pandemic in 2020 and 2021. Geneva: World Health Organization; 2022. Available from: https://www.who.int/news/item/05-05-2022-14.9-million-excess-deaths-were-associated-with-the-covid-19-pandemic-in-2020-and-2021 [cited 2024 Feb 6].

[2] Cutler DM, Summers LH. The COVID-19 pandemic and the $16 trillion virus. JAMA. 2020 Oct 20;324(15):1495–6. 10.1001/jama.2020.19759 33044484 PMC7604733

[3] Mazzucato M. Health for all: transforming economies to deliver what matters. BMJ. 2023 May 23;381:1175. 10.1136/bmj.p1175 37220921

[4] Health for all: transforming economies to deliver what matters: final report of the WHO Council on the Economics of Health for All. Geneva: World Health Organization; 2023. Available from: https://iris.who.int/handle/10665/373122 [cited 2024 Feb 6].

[5] The WHO Council on the Economics of Health for All. Valuing health for all: rethinking and building a whole-of-society approach. Council Brief No. 3. Geneva: World Health Organization; 2022. Available from: https://cdn.who.int/media/docs/default-source/council-on-the-economics-of-health-for-all/who_councilbrief3.pdf?sfvrsn=b121f943_11&download=true [cited 2024 Feb 6].

[6] Closing the gap in a generation: health equity through action on the social determinants of health - Final report of the commission on social determinants of health. Geneva: World Health Organization; 2008. Available from: https://www.who.int/publications/i/item/WHO-IER-CSDH-08.1 [cited 2024 Feb 6].

[7] Macroeconomics and health: investing in health for economic development. Report of the Commission on Macroeconomics and Health. Geneva: World Health Organization; 2001. Available from: https://www.who.int/publications/i/item/924154550X [cited 2024 Feb 6].

[8] Vanoli A. On the report by the Commission on the Measurement of Economic Performance and Social Progress (2009). RatSWD Working Paper No. 62; 2010. Available from: 10.2139/ssrn.1714428 [cited 2024 Feb 6]. 10.2139/ssrn.1714428

[9] Analysis of pandemic preparedness and response (PPR) architecture and financing needs and gaps [internet]. Washington, DC and Geneva: World Bank and World Health Organization; 2022. Available from: https://thedocs.worldbank.org/en/doc/5760109c4db174ff90a8dfa7d025644a-0290032022/original/G20-Gaps-in-PPR-Financing-Mechanisms-WHO-and-WB-pdf.pdf [cited 2024 Feb 6].

[10] Glassman A, Smitham E. The next pandemic could come soon and be deadlier. Washington, DC: Center for Global Development; 2021. Available from: https://www.cgdev.org/blog/the-next-pandemic-could-come-soon-and-be-deadlier [cited 2024 Feb 6].

[11] Peters MD, Godfrey CM, Khalil H, McInerney P, Parker D, Soares CB. Guidance for conducting systematic scoping reviews. Int J Evid Based Healthc. 2015 Sep;13(3):141–6. 10.1097/XEB.0000000000000050 26134548

[12] Angeli F, Camporesi S, Dal Fabbro G. The COVID-19 wicked problem in public health ethics: conflicting evidence, or incommensurable values? Humanit Soc Sci Commun. 2021;8(1):161. 10.1057/s41599-021-00839-1

[13] Duran-Fernandez R, Bernal-Serrano D, Garcia-Huitron A, Hutubessy RCW. Pandemic preparedness and response financing: a critical systematic review. INPLASY202380111. Middletown: International Platform of Registered Systematic Review and Meta-analysis Protocols; 2023. Available from: https://inplasy.com/inplasy-2023-8-0111/ [cited 2024 Feb 6]. 10.37766/inplasy2023.8.0111

[14] Page MJ, McKenzie JE, Bossuyt PM, Boutron I, Hoffmann TC, Mulrow CD, et al. The PRISMA 2020 statement: an updated guideline for reporting systematic reviews. Int J Surg. 2021 Apr;88:105906. 10.1016/j.ijsu.2021.105906 33789826

[15] Tricco AC, Lillie E, Zarin W, O’Brien KK, Colquhoun H, Levac D, et al. PRISMA extension for scoping reviews (PRISMA-ScR): checklist and explanation. Ann Intern Med. 2018 Oct 2;169(7):467–73. 10.7326/M18-0850 30178033

[16] Williams V, Boylan AM, Nunan D. Critical appraisal of qualitative research: necessity, partialities and the issue of bias. BMJ Evid Based Med. 2020 Feb;25(1):9–11. 10.1136/bmjebm-2018-111132 30862711

[17] Scimago Journal & Country Rank [internet]. Granada: Scimago Lab; 2024. Available from: https://www.scimagojr.com [cited 2024 Feb 6].

[18] Daems R, Del Giudice G, Rappuoli R. Anticipating crisis: towards a pandemic flu vaccination strategy through alignment of public health and industrial policy. Vaccine. 2005 Dec 30;23(50):5732–42. 10.1016/j.vaccine.2005.10.011 16271423

[19] Gostin LO, Berkman BE. Pandemic influenza: ethics, law, and the public’s health. Washington, DC: Georgetown Law Faculty publications and other works; 2007. Available at: https://scholarship.law.georgetown.edu/facpub/449/ [cited 2024 Feb 6].

[20] Ijsselmuiden C, Matlin SA, Maïga AH, Hasler J, Pannenborg O, Evans T, et al. Steering Committee of the 2008 Global Ministerial Forum on Research for Health. From Mexico to Mali: a new course for global health. Lancet. 2008 Jan 12;371(9607):91–3. 10.1016/S0140-6736(08)60080-X 18191667

[21] Ortu G, Mounier-Jack S, Coker R. Pandemic influenza preparedness in Africa is a profound challenge for an already distressed region: analysis of national preparedness plans. Health Policy Plan. 2008 May;23(3):161–9. 10.1093/heapol/czn004 18381384 PMC7314001

[22] Yen C, Hyde TB, Costa AJ, Fernandez K, Tam JS, Hugonnet S, et al. The development of global vaccine stockpiles. Lancet Infect Dis. 2015 Mar;15(3):340–7. 10.1016/S1473-3099(14)70999-5 25661473 PMC4712379

[23] Katz R, Seifman R. Opportunities to finance pandemic preparedness. Lancet Glob Health. 2016 Nov;4(11):e782–3. 10.1016/S2214-109X(16)30202-9 27692862

[24] Leigh J, Fitzgerald G, Garcia E, Moon S. Global epidemics: how well can we cope? BMJ. 2018 Aug 9;362:k3254. 10.1136/bmj.k3254 30101744

[25] Babu GR, Khetrapal S, John DA, Deepa R, Narayan KMV. Pandemic preparedness and response to COVID-19 in South Asian countries. Int J Infect Dis. 2021 Mar;104:169–74. 10.1016/j.ijid.2020.12.048 33370566 PMC7836380

[26] Charani E, McKee M, Ahmad R, Balasegaram M, Bonaconsa C, Merrett GB, et al. Optimising antimicrobial use in humans - review of current evidence and an interdisciplinary consensus on key priorities for research. Lancet Reg Health Eur. 2021 Jun 29;7:100161. 10.1016/j.lanepe.2021.100161 34557847 PMC8454847

[27] Duff JH, Liu A, Saavedra J, Batycki JN, Morancy K, Stocking B, et al. A global public health convention for the 21st century. Lancet Public Health. 2021 Jun 6;6:e428–e33. 10.1016/S2468-2667(21)00070-0 33964227 PMC8099565

[28] Giersing B, Shah N, Kristensen D, Amorij JP, Kahn AL, Gandrup-Marino K, et al. Strategies for vaccine-product innovation: creating an enabling environment for product development to uptake in low- and middle-income countries. Vaccine. 2021 Dec 3;39(49):7208–19. 10.1016/j.vaccine.2021.07.091 34627624 PMC8657812

[29] Kleinert S, Horton R. Can COVID-19 help accelerate and transform the diagnostics agenda? Lancet. 2021 Nov 27;398(10315):1945–7. 10.1016/S0140-6736(21)02093-6 34626541 PMC8494466

[30] Lal A, Erondu NA, Heymann DL, Gitahi G, Yates R. Fragmented health systems in COVID-19: rectifying the misalignment between global health security and universal health coverage. Lancet. 2021 Jan 2;397(10268):61–7. 10.1016/S0140-6736(20)32228-5 33275906 PMC7834479

[31] Lurie N, Keusch GT, Dzau VJ. Urgent lessons from COVID-19: why the world needs a standing, coordinated system and sustainable financing for global research and development. Lancet. 2021 Mar 27;397(10280):1229–36. 10.1016/S0140-6736(21)00503-1 33711296 PMC7993931

[32] Sirleaf EJ, Clark H. Report of the Independent Panel for Pandemic Preparedness and Response: making COVID-19 the last pandemic. Lancet. 2021 Jul 10;398(10295):101–3. 10.1016/S0140-6736(21)01095-3 33991477 PMC9751704

[33] Agarwal R, Reed T. Financing vaccine equity: funding for day-zero of the next pandemic. Oxf Rev Econ Policy. 2022;38(4):833–50. 10.1093/oxrep/grac032

[34] Akenroye TO, Abubakre A, Elbaz J, Vishnu CR, Beka Be Nguema JN, Rana G, et al. Modeling the barriers to multistakeholder collaboration for COVID-19 pandemic response: evidence from Sub-Saharan Africa. Int Public Manage J. 2022;25(2):192–216. 10.1080/10967494.2021.1970061

[35] Bollyky TJ, Hulland EN, Barber RM, Collins JK, Kiernan S, Moses M, et al. COVID-19 National Preparedness Collaborators. Pandemic preparedness and COVID-19: an exploratory analysis of infection and fatality rates, and contextual factors associated with preparedness in 177 countries, from Jan 1, 2020, to Sept 30, 2021. Lancet. 2022 Apr 16;399(10334):1489–512. 10.1016/S0140-6736(22)00172-6 35120592 PMC8806194

[36] Carlson CJ, Phelan AL. International law reform for One Health notifications. Lancet. 2022 Aug 6;400(10350):462–8. 10.1016/S0140-6736(22)00942-4 35810748

[37] Cernuschi T, Malvolti S, Hall S, Debruyne L, Bak Pedersen H, Rees H, et al. The quest for more effective vaccine markets - opportunities, challenges, and what has changed with the SARS-CoV-2 pandemic. Vaccine. 2022 Oct 21;S0264-410X(22)00920-3. 10.1016/j.vaccine.2022.07.032 38103962 PMC9585501

[38] Clark H, Cárdenas M, Dybul M, Kazatchkine M, Liu J, Miliband D, et al. Transforming or tinkering: the world remains unprepared for the next pandemic threat. Lancet. 2022 May 28;399(10340):1995–9. 10.1016/S0140-6736(22)00929-1 35597246 PMC9114832

[39] Frenk J, Godal T, Gómez-Dantés O, Store JG. A reinvigorated multilateralism in health: lessons and innovations from the COVID-19 pandemic. Lancet. 2022 Nov 5;400(10363):1565–8. 10.1016/S0140-6736(22)01943-2 36216020 PMC9544940

[40] Gwenzi W, Skirmuntt EC, Musvuugwa T, Teta C, Halabowski D, Rzymski P. Grappling with (re)-emerging infectious zoonoses: risk assessment, mitigation framework, and future directions. Int J Disaster Risk Reduct. 2022 Nov;82:103350. 10.1016/j.ijdrr.2022.103350

[41] Hayman B, Kumar Suri R, Downham M. Sustainable vaccine manufacturing in low- and middle-income countries. Vaccine. 2022 Nov 28;40(50):7288–304. 10.1016/j.vaccine.2022.10.044 36334966

[42] Lal A, Abdalla SM, Chattu VK, Erondu NA, Lee TL, Singh S, et al. Pandemic preparedness and response: exploring the role of universal health coverage within the global health security architecture. Lancet Glob Health. 2022 Nov;10(11):e1675–83. 10.1016/S2214-109X(22)00341-2 36179734 PMC9514836

[43] Meier BM, Habibi R, Gostin LO. A global health law trilogy: transformational reforms to strengthen pandemic prevention, preparedness, and response. J Law Med Ethics. 2022;50(3):625–7. 10.1017/jme.2022.103 36398645

[44] Moeti M, Gao GF, Herrman H. Global pandemic perspectives: public health, mental health, and lessons for the future. Lancet. 2022 Aug 27;400(10353):e3–7. 10.1016/S0140-6736(22)01328-9 35934013 PMC9352273

[45] Olliaro P, Torreele E. Global challenges in preparedness and response to epidemic infectious diseases. Mol Ther. 2022 May 4;30(5):1801–9. 10.1016/j.ymthe.2022.02.022 35218930 PMC8864962

[46] Reid-Henry S, Lidén J, Benn C, Saminarsih D, Herlinda O, Venegas MFB. A new paradigm is needed for financing the pandemic fund. Lancet. 2022 Jul 30;400(10349):345–6. 10.1016/S0140-6736(22)01239-9 35779554

[47] Ren M, So AD, Chandy SJ, Mpundu M, Peralta AQ, Åkerfeldt K, et al. Equitable access to antibiotics: a core element and shared global responsibility for pandemic preparedness and response. J Law Med Ethics. 2022;50(S2):34–9. 10.1017/jme.2022.77 36889350 PMC10009365

[48] Rosa WE, Ahmed E, Chaila MJ, Chansa A, Cordoba MA, Dowla R, et al. Can you hear us now? Equity in global advocacy for palliative care. J Pain Symptom Manage. 2022 Oct;64(4):e217–26. 10.1016/j.jpainsymman.2022.07.004 35850443 PMC9482940

[49] Sachs JD, Karim SSA, Aknin L, Allen J, Brosbøl K, Colombo F, et al. The Lancet Commission on lessons for the future from the COVID-19 pandemic. Lancet. 2022 Oct 8;400(10359):1224–80. 10.1016/S0140-6736(22)01585-9 36115368 PMC9539542

[50] Saxenian H, Alkenbrack S, Freitas Attaran M, Barcarolo J, Brenzel L, Brooks A, et al. Sustainable financing for Immunization Agenda 2030. Vaccine. 2022 Dec 1;40(48):7375–6. 10.1016/j.vaccine.2022.11.037 36464542

[51] Sekalala S, Rawson B. The role of civil society in mobilizing human rights struggles for essential medicines: a critique from HIV/AIDS to COVID-19. Health Hum Rights. 2022 Dec;24(2):177–89. 36579304 PMC9790953

[52] Solomon S. Challenges and prospects for the intergovernmental negotiations to develop a new instrument on pandemic prevention, preparedness, and response. J Law Med Ethics. 2022;50(4):860–3. 10.1017/jme.2023.29 36883391

[53] Tacconelli E, Gorska A, Carrara E, Davis RJ, Bonten M, Friedrich AW, et al. Challenges of data sharing in European COVID-19 projects: a learning opportunity for advancing pandemic preparedness and response. Lancet Reg Health Eur. 2022 Oct;21:100467. 10.1016/j.lanepe.2022.100467 35942201 PMC9351292

[54] Williamson A, Forman R, Azzopardi-Muscat N, Battista R, Colombo F, Glassman A, et al. Effective post-pandemic governance must focus on shared challenges. Lancet. 2022 May 28;399(10340):1999–2001. 10.1016/S0140-6736(22)00891-1 35588759 PMC9754057

[55] Akselrod S, Barron GC, Dain K, Ditiu L, Fogstad H, Karema C, et al. Getting health back on the highest political agenda – the UN High-level Meetings on health in 2023. Lancet Glob Health. 2023 Jun;11(6):e819–20. 10.1016/S2214-109X(23)00166-3 37001539 PMC10188362

[56] Archer BN, Abdelmalik P, Cognat S, Grand PE, Mott JA, Pavlin BI, et al. Defining collaborative surveillance to improve decision making for public health emergencies and beyond. Lancet. 2023 Jun 3;401(10391):1831–4. 10.1016/S0140-6736(23)01009-7 37230104 PMC10202415

[57] Bochner AF, Makumbi I, Aderinola O, Abayneh A, Jetoh R, Yemanaberhan RL, et al. Implementation of the 7-1-7 target for detection, notification, and response to public health threats in five countries: a retrospective, observational study. Lancet Glob Health. 2023 Jun;11(6):e871–9. 10.1016/S2214-109X(23)00133-X 37060911 PMC10156425

[58] Boyce MR, Sorrell EM, Standley CJ. An early analysis of the World Bank’s Pandemic Fund: a new fund for pandemic prevention, preparedness and response. BMJ Glob Health. 2023 Jan;8(1):e011172. 10.1136/bmjgh-2022-011172 36599499 PMC9815014

[59] Byanyima W, Lauterbach K, Kavanagh MM. Community pandemic response: the importance of action led by communities and the public sector. Lancet. 2023 Jan 28;401(10373):253–5. 10.1016/S0140-6736(22)02575-2 36528036 PMC9750179

[60] Elnaiem A, Mohamed-Ahmed O, Zumla A, Mecaskey J, Charron N, Abakar MF, et al. Global and regional governance of One Health and implications for global health security. Lancet. 2023 Feb 25;401(10377):688–704. 10.1016/S0140-6736(22)01597-5 36682375

[61] Ford A, Hwang A, Mo AX, Baqar S, Touchette N, Deal C, et al. Meeting Summary: Global Vaccine and Immunization Research Forum, 2021. Vaccine. 2023 Mar 10;41(11):1799–807. 10.1016/j.vaccine.2023.02.028 36803897 PMC9938725

[62] Gallo-Cajiao E, Lieberman S, Dolšak N, Prakash A, Labonté R, Biggs D, et al. Global governance for pandemic prevention and the wildlife trade. Lancet Planet Health. 2023 Apr;7(4):e336–45. 10.1016/S2542-5196(23)00029-3 37019574 PMC10069821

[63] Gostin LO, Friedman EA, Hossain S, Mukherjee J, Zia-Zarifi S, Clinton C, et al. Human rights and the COVID-19 pandemic: a retrospective and prospective analysis. Lancet. 2023 Jan 14;401(10371):154–68. 10.1016/S0140-6736(22)01278-8 36403583 PMC9671650

[64] Helldén D, Tesfaye S, Gachen C, Lindstrand A, Källander K. Digital health funding for COVID-19 vaccine deployment across four major donor agencies. Lancet Digit Health. 2023 Sep;5(9):e627–31. 10.1016/S2589-7500(23)00134-6 37625897

[65] Hiroshima G7 Global Health Task Force. Promote global solidarity to advance health-system resilience: proposals for the G7 meetings in Japan. Lancet. 2023 Apr 22;401(10385):1319–21. 10.1016/S0140-6736(23)00690-6 37028441

[66] Kasaeva T, Dias HM, Pai M. Fast-tracking progress to end TB: high-level opportunities for investment and action. Lancet. 2023 Mar 25;401(10381):975–8. 10.1016/S0140-6736(23)00460-9 36958358

[67] Katz R. Challenges of tracking funding for pandemic preparedness and response. Lancet Glob Health. 2023 Mar;11(3):e310–1. 10.1016/S2214-109X(23)00017-7 36706769 PMC9873267

[68] Khosla R, McCoy D, Marriot A. Backsliding on human rights and equity in the Pandemic Accord. Lancet. 2023 Jun 17;401(10393):2019–21. 10.1016/S0140-6736(23)01118-2 37271154

[69] Lee TL. Realising the right to participate in pandemic prevention, preparedness and response and beyond. BMJ Glob Health. 2023 Jan;8(1):e011689. 10.1136/bmjgh-2023-011689 36707094 PMC9884853

[70] Micah AE, Bhangdia K, Cogswell IE, Lasher D, Lidral-Porter B, Maddison ER, et al. Global Burden of Disease 2021 Health Financing Collaborator Network. Global investments in pandemic preparedness and COVID-19: development assistance and domestic spending on health between 1990 and 2026. Lancet Glob Health. 2023 Mar;11(3):e385–413. 10.1016/S2214-109X(23)00007-4 36706770 PMC9998276

[71] Morris S. Development finance cooperation amidst great power competition: what role for the World Bank? Oxf Rev Econ Policy. 2023;39(2):379–88. 10.1093/oxrep/grad006

[72] Müller SA, Agweyu A, Akanbi OA, Alex-Wele MA, Alinon KN, Arora RK, et al. Public Health Collaborators on Serosurveillance for Pandemic Preparedness and Response PHSeroPPR. Learning from serosurveillance for SARS-CoV-2 to inform pandemic preparedness and response. Lancet. 2023 Jul 29;402(10399):356–8. 10.1016/S0140-6736(23)00964-9 37247625 PMC10219629

[73] Nardi F, Ginsbach K, Aneja K, Gottschalk K, Halabi S. COVID-19 in the Americas: the role of collaborating centers in understanding lessons and best practices in pandemic preparedness and response. Rev Panam Salud Publica. 2023 Mar 10;47:e7. 10.26633/RPSP.2023.7 36909808 PMC9976272

[74] Saxena A, Baker BK, Banda A, Herlitz A, Miller J, Karrar K, et al. Pandemic preparedness and response: beyond the Access to COVID-19 Tools Accelerator. BMJ Glob Health. 2023 Jan;8(1):e010615. 10.1136/bmjgh-2022-010615 36650015 PMC9852735

[75] Stubbs T, Kentikelenis A, Gabor D, Ghosh J, McKee M. The return of austerity imperils global health. BMJ Glob Health. 2023 Feb;8(2):e011620. 10.1136/bmjgh-2022-011620 36804732 PMC9944267

[76] Torreele E, Kazatchkine M, Liu J, Dybul M, Cárdenas M, Singh S, et al. Stopping epidemics when and where they occur. Lancet. 2023 Feb 4;401(10374):324–8. 10.1016/S0140-6736(23)00015-6 36642089 PMC9836401

[77] Torreele E, McNab C, Adeyi O, Bonnell R, Dhaliwal M, Hassan F, et al. It is time for ambitious, transformational change to the epidemic countermeasures ecosystem. Lancet. 2023 Mar 25;401(10381):978–82. 10.1016/S0140-6736(23)00526-3 36924776

[78] Hirsch JE, Buela-Casal G. The meaning of the h-index. Int J Clin Health Psychol. 2014;14(2):161–4. 10.1016/S1697-2600(14)70050-X

[79] Pandemic prevention, preparedness and response accord. Geneva: World Health Organization; 2023. Available from: https://www.who.int/news-room/questions-and-answers/item/pandemic-prevention--preparedness-and-response-accord [cited 2024 Feb 6].

[80] New fund for pandemic prevention, preparedness and response formally established. Washington, DC: The World Bank Group; 2023. Available from: https://www.worldbank.org/en/news/press-release/2022/09/09/new-fund-for-pandemic-prevention-preparedness-and-response-formally-established [cited 2024 Feb 6].

[81] Fact sheet: White House launches Office of Pandemic Preparedness and Response Policy. Washington, DC: The White House; 2023. Available from: https://www.whitehouse.gov/briefing-room/statements-releases/2023/07/21/fact-sheet-white-house-launches-office-of-pandemic-preparedness-and-response-policy/ [cited 2024 Feb 6].

[82] Mahood Q, Van Eerd D, Irvin E. Searching for grey literature for systematic reviews: challenges and benefits. Res Synth Methods. 2014 Sep;5(3):221–34. 10.1002/jrsm.1106 26052848

[83] Corlett RT. Trouble with the gray literature. Biotropica. 2011;43(1):3–5. 10.1111/j.1744-7429.2010.00714.x

[84] Paez A. Gray literature: An important resource in systematic reviews. J Evid Based Med. 2017 Aug;10(3):233–40. 10.1111/jebm.12266 28857505

[85] Kousha K, Thelwall M, Bickley M. The high scholarly value of grey literature before and during COVID-19. Scientometrics. 2022;127(6):3489–504. 10.1007/s11192-022-04398-3 35615527 PMC9122808

[86] Duran-Fernandez R, Garcia-Huitron A. ¿Qué falló en el manejo de la pandemia de COVID-19? Este Pais. 2022;378(Nov). Spanish. Available from: https://estepais.com/tendencias_y_opiniones/manejo-pandemia-covid-19/ [cited 2023 Dec 1].

[87] Evaborhene NA, Udokanma EE, Adebisi YA, Okorie CE, Kafuko Z, Conde HM, et al. The Pandemic Treaty, the Pandemic Fund, and the Global Commons: our scepticism. BMJ Global Health. 2023;8:e011431. . 10.1136/bmjgh-2022-011431 36854490 PMC9980354

[88] Stein F, Sridhar D. Health as a “global public good”: creating a market for pandemic risk. BMJ. 2017 Aug 31;358:j3397. 10.1136/bmj.j3397 28860109 PMC5594412

[89] Hiscott J, Alexandridi M, Muscolini M, Tassone E, Palermo E, Soultsioti M, et al. The global impact of the coronavirus pandemic. Cytokine Growth Factor Rev. 2020 Jun;53:1–9. 10.1016/j.cytogfr.2020.05.010 32487439 PMC7254014

[90] The sustainable development goals report 2023: special edition. New York: United Nations; 2023. Available from: https://unstats.un.org/sdgs/report/2023/ [cited 2024 Feb 6].

[91] Proposal to establish a Resilience and Sustainability Trust. IMF policy paper. Washington, DC: International Monetary Fund; 2022. Available from: https://www.elibrary.imf.org/downloadpdf/journals/007/2022/013/007.2022.issue-013-en.pdf [cited 2024 Feb 6].

